# A machine learning–Based model to predict early death among bone metastatic breast cancer patients: A large cohort of 16,189 patients

**DOI:** 10.3389/fcell.2022.1059597

**Published:** 2022-12-07

**Authors:** Fan Xiong, Xuyong Cao, Xiaolin Shi, Ze Long, Yaosheng Liu, Mingxing Lei

**Affiliations:** ^1^ Department of Orthopedic Surgery, People’s Hospital of Macheng City, Huanggang, China; ^2^ Department of Orthopedic Surgery, The Fifth Medical Center of Chinese PLA General Hospital, Beijing, China; ^3^ Department of Orthopedic Surgery, The Second Affiliated Hospital of Zhejiang Chinese Medical University, Hangzhou, China; ^4^ Department of Orthopedics, The Second Xiangya Hospital of Central South University, Changsha, China; ^5^ Senior Department of Orthopedics, The Fourth Medical Center of PLA General Hospital, Beijing, China; ^6^ Department of Orthopedic Surgery, National Clinical Research Center for Orthopedics, Sports Medicine, and Rehabilitation, Beijing, China; ^7^ Department of Orthopedic Surgery, Hainan Hospital of PLA General Hospital, Sanya, China; ^8^ Chinese PLA Medical School, Beijing, China

**Keywords:** breast cancer, early death, machine learning, prediction model, bone metastasis

## Abstract

**Purpose:** This study aims to develop a prediction model to categorize the risk of early death among breast cancer patients with bone metastases using machine learning models.

**Methods:** This study examined 16,189 bone metastatic breast cancer patients between 2010 and 2019 from a large oncological database in the United States. The patients were divided into two groups at random in a 90:10 ratio. The majority of patients (*n* = 14,582, 90%) were served as the training group to train and optimize prediction models, whereas patients in the validation group (*n* = 1,607, 10%) were utilized to validate the prediction models. Four models were introduced in the study: the logistic regression model, gradient boosting tree model, decision tree model, and random forest model.

**Results:** Early death accounted for 17.4% of all included patients. Multivariate analysis demonstrated that older age; a separated, divorced, or widowed marital status; nonmetropolitan counties; brain metastasis; liver metastasis; lung metastasis; and histologic type of unspecified neoplasms were significantly associated with more early death, whereas a lower grade, a positive estrogen receptor (ER) status, cancer-directed surgery, radiation, and chemotherapy were significantly the protective factors. For the purpose of developing prediction models, the 12 variables were used. Among all the four models, the gradient boosting tree had the greatest AUC [0.829, 95% confident interval (CI): 0.802–0.856], and the random forest (0.828, 95% CI: 0.801–0.855) and logistic regression (0.819, 95% CI: 0.791–0.847) models came in second and third, respectively. The discrimination slopes for the three models were 0.258, 0.223, and 0.240, respectively, and the corresponding accuracy rates were 0.801, 0.770, and 0.762, respectively. The Brier score of gradient boosting tree was the lowest (0.109), followed by the random forest (0.111) and logistic regression (0.112) models. Risk stratification showed that patients in the high-risk group (46.31%) had a greater six-fold chance of early death than those in the low-risk group (7.50%).

**Conclusion:** The gradient boosting tree model demonstrates promising performance with favorable discrimination and calibration in the study, and this model can stratify the risk probability of early death among bone metastatic breast cancer patients.

## Introduction

Breast cancer poses a serious threat to the global health problem with an estimated 2.3 million new cases (11.7%) in 2020, ranking as the most commonly diagnosed malignancy among female patients (Sung et al., 2021). In addition, breast cancer is the leading cause of cancer death in women, accounting for 0.7 million new cancer-related deaths in 2020 (Sung et al., 2021), and it ranks the fifth in terms of mortality among all cancer patients. Besides, breast cancer incidence continues to rise with the mortality decreasing slightly mainly due to early detection, greater knowledge, and therapeutic improvements (Siegel et al., 2022). However, survival prognosis is far from satisfactory, especially among less developed countries because of delayed diagnosis and a lack of access to effective treatments (Kashyap et al., 2022; Wilkinson and Gathani, 2022). Therefore, an increasing global burden of breast cancer is inevitable (Wilkinson and Gathani, 2022).

Bone is the most frequent site for breast cancer metastases, developing in 65.0%–80% of patients (Kuchuk et al., 2013; Body et al., 2017). Of note, bone metastatic breast cancer is an advanced stage and characterized by pathologic fracture, spinal cord compression, endocrine dysregulation, and increased mobility, which has a detrimental effect on the patient’s survival outcome, which worsens the patient’s quality of life (Brook et al., 2018). It has been reported that the median survival time for breast cancer patients with bone metastases is about 2.0 years (Pan et al., 2021), and half the number of breast cancer patients treated with surgery for bone metastases die within 30 months (Mou et al., 2021).

Currently, there is no therapeutic benchmark for the management of bone metastases in breast cancer, which brings challenges to both patients and physicians. Surgical interventions of bone metastatic breast cancer patients typically include minimal invasive surgery and open surgery, such as stabilization or replacement of the destructive bone. The basic objective of any treatment is to maximize patient’s functional outcome and improve the quality of life among those patients (Hankins et al., 2021). In this context, prediction of early death is critical for such patients, because therapeutic strategies are conducted largely depending on the accurate and personalized prediction of life span (Kirkinis et al., 2016). Generally, patients with longer life expectancies should be treated with more aggressive treatments like invasive surgery of tumor excision in the long bone and spine or relatively long-course radiotherapy (Tsukamoto et al., 2021), whereas patients with shorter life expectancies are recommended to receive the best supportive care and minimal invasive surgery like vertebroplasty or short-course radiotherapy (Tsukamoto et al., 2021). Inappropriate estimation of survival may lead to over- or under-treatments, which can accelerate patient death or result in a low quality of life.

Therefore, the aim of our study was to develop a reliable prediction model that would explicitly stratify the risk of early death among bone metastatic breast cancer patients. In this study, the logistic regression model and three machine learning models were introduced and compared in order to improve the accuracy of prediction. We found that the gradient boosting tree model performed promisingly and could stratify the risk probability of early death in bone metastatic breast cancer patients.

## Patients and methods

### Patients and study design

The data for this study, which examined 23,045 breast cancer patients with bone metastases between 2010 and 2019, were taken from the Surveillance, Epidemiology, and End Results (SEER) database, which can be accessed at https://seer.cancer.gov. According to SEER, an authoritative data source for cancer statistics in the United States, the cancer incidence and population information were broken down by age, sex, race, year of diagnosis, and geographic regions. In addition, the SEER database updates its research data each spring depending on the previous November’s submission of data. The database can be accessed publicly and provides patient data without requiring personal identification, so ethical approval and informed permissions were not necessary. Using the reference number 23489-Nov2020, we were given permission to access the database of the National Cancer Institute in the United States. The human data were in accordance with the Declaration of Helsinki.

**FIGURE 1:**
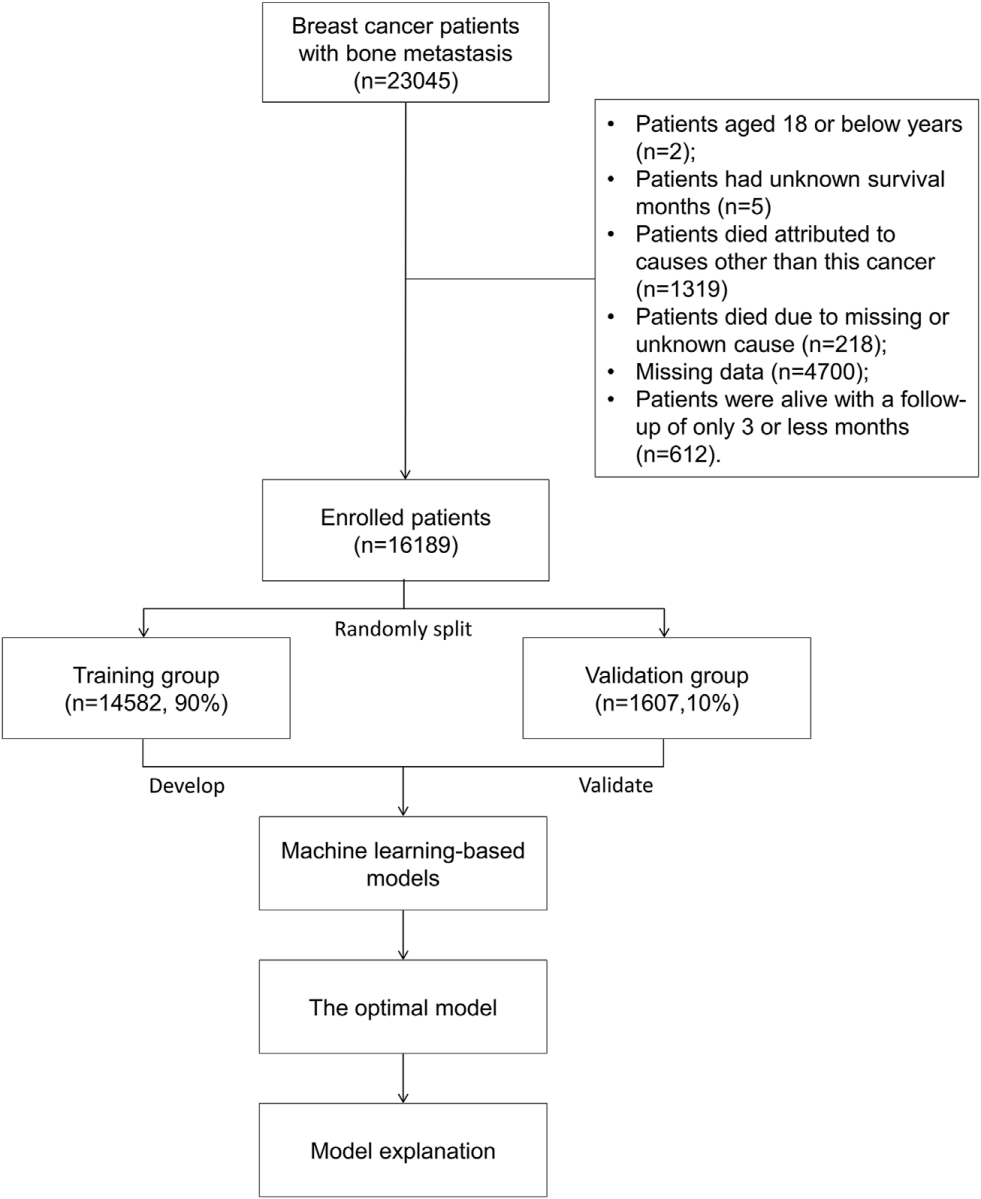
Flowchart of the study.

For analysis, patients with breast cancer with bone metastases were included. The following were the exclusion criteria: patients aged 18 years or younger (Sung et al., 2021); patients having no recorded survival time (Siegel et al., 2022); patients who died from causes other than this cancer (Wilkinson and Gathani, 2022); patients who died from causes that were unknown or missing (Kashyap et al., 2022); patients with missing data (Kuchuk et al., 2013); patients who were alive with a follow-up of 3 months or less (Body et al., 2017). At last, based on the aforementioned criteria, 16,189 individuals with bone metastatic breast cancer were enrolled for analysis. A training group (*n* = 14,582, 90%) and validation group (*n* = 1,607, 10%) were randomly developed from the entire patient cohort. Figure 1 shows patients’ flowchart.

### Extraction of characteristics

The clinical characteristics for analysis in the study included patients' demographics, cancer stages, metastatic conditions, hormone status, therapeutic strategies, and survival times. The patient demographics included age (years), sex (female *vs*. male), race (American Indian/Alaska Native *vs*. Asian or Pacific Islander *vs*. black *vs*. white *vs*. unknown), marital status [married (which includes common law) *vs*. separated, divorced, or widowed *vs*. single (never married) *vs*. others], and geographic areas (metropolitan counties *vs*. nonmetropolitan counties *vs*. unknown). Cancer-related information included laterality (bilateral, single primary *vs*. left—origin of primary *vs*. only one side—side unspecified *vs*. paired site laterality, but no information concerning laterality *vs*. right—origin of primary), histologic type [adenomas and adenocarcinomas *vs*. ductal and lobular neoplasms *vs*. epithelial neoplasms (not otherwise specified, NOS) *vs*. squamous cell neoplasms *vs*. unspecified neoplasms *vs*. others], grade (I *vs*. II *vs*. III *vs*. IV *vs*. Unknown), T stage (T0 *vs*. T1 *vs*. T2 *vs*. T3 *vs*. T4 *vs*. TX), and N stage (N0 *vs*. N1 *vs*. N2 *vs*. N3 *vs*. NX). The presence of metastatic conditions included brain metastasis (no *vs*. unknown *vs*. yes), liver metastasis (no *vs*. unknown *vs*. yes), and lung metastasis (no *vs*. unknown *vs*. yes). In terms of hormone state, there were estrogen receptor (ER) status (borderline/unknown *vs*. negative *vs*. positive), progesterone receptor (PR) status (borderline/unknown *vs*. negative *vs*. positive), and human epidermal growth factor receptor-2 (HER_2_) (borderline/unknown *vs*. negative *vs*. positive). Cancer therapeutic approaches included cancer-directed surgery (no *vs*. unknown *vs*. yes), radiation (no/unknown *vs*. yes), and chemotherapy (no/unknown *vs*. yes). Age was patient’s age at cancer diagnosis. Early death in the study was defined as patients who died within or at 3 months, and survival outcome referred to cancer-specific survival.

### Model development and estimation

Selection of significant risk factors for early death was achieved by utilizing multiple logistic regressions. Models were developed with significant variables that had a *p*-value of less than 0.01. Four models—the logistic regression, gradient boosting tree, decision tree, and random forest—were introduced to train and optimize models in the training group. The most effective model super-parameters were discovered *via* grid search or random hyper-parameter search.

In the validation group, measures of prediction performance were used to evaluate models, and these measures included mean predicted probability, Brier score, intercept, calibration slope, area under the curve (AUC), discrimination slope, specificity, sensitivity (recall), negative predictive value (NPV), positive predictive value (PPV, precision), Youden index, and accuracy. The Brier score was the mean squared error between the actual outcome and estimated risk (Huang et al., 2020), as shown in the below equation:

$$Brier\;score=\frac{\sum_{i=1}^{N}{\left(E_{i}-O_{i}\right)}^{2}}{N}.$$

Here, N stands for the number of patients, $E_{i}$ for the predicted risk for patients i , and $O_{i}$ for the actual outcome for patients i . Since it includes components of both discrimination and calibration (Rufibach, 2010), the Brier score is a metric that is used to evaluate overall prediction ability of models, with lower values indicating better calibration. A Brier score of more than 0.25 suggests a useless model. The calibration slope is determined by comparing the predicted probability of early death against the actual probability of early death in calibration curves (Steyerberg and Vergouwe, 2014). The AUC is a crucial metric to evaluate the model’s capacity for discrimination. AUC values of greater than 0.80 represent favorable discrimination. The discrimination slope is the mean difference of predicted probability between patients with and without early death (Pencina et al., 2017). Regarding the evaluation of specificity, sensitivity, NPV, and PPV, confusion matrix was used for analysis (Supplementary Figure S1). The Youden index is the sum of sensitivity and specificity, with a larger value indicating better performance of models. In addition, the decision curve analysis was used to evaluate the model’s clinical usefulness through calculating the net benefits in a range of threshold probabilities. After thoroughly evaluating each model’s ability to predict, the best model could be identified.

### Model explainability and risk stratification

The optimal model was used to present model explainability using the SHapley Additive exPlanations (SHAP). Furthermore, the variable importance was summarized on the basis of the contributions to early death in the study. In terms of the threshold of the optimal model, the risk stratification was carried out. To be specific, patients were divided into two groups: those with a predicted probability of less than the threshold and those with a predicted probability of more than the threshold, referred to as the low-risk and high-risk groups, respectively. A Kaplan–Meier survival curve was plotted among patients stratified by the two risk groups, and the difference between the two risk groups was compared using the log-rank test.

### Statistical evaluation

While the quantitative variables were presented as mean ± standard deviation (SD), the qualitative variables were depicted as proportions. The comparison of the quantitative variables was achieved using the *t* tests, and the comparison of the qualitative variables was achieved using the Chi-square tests and adjusted continuity Chi-square tests. Python (version 3.9.7) was used to perform modelling analysis, model explanation, and variable importance, and the R programming language (version 4.1.2) (https://www.r-project.org/) was used to carry out the statistical analysis. The significance level was set at 0.05 (two-tailed sides).

## Results

### Patients' demographics and clinical characteristics

A total of 16,189 patients were enrolled for analysis in the study. The mean age was 61.67 ± 14.04 years. The majority of the patients (98.8%) were female, 76.9% were white, 43.6% were married, and 88.6% were from metropolitan counties. Regarding organ metastasis, the lung (26.0%) was the most common site, and this was followed by the liver (23.0%) and brain (7.3%). Ductal and lobular neoplasms represented the most typical histologic type (83.7%). Patients’ T stage, N stage, and tumor grade are shown in Table 1. As for the hormone status, a multitude of patients were ER^+^ (76.1%), PR^+^ (61.2%), and HER2^−^ (67.2%). Cancer-directed surgery, radiation, and chemotherapy accounted for 22.4%, 33.8%, and 52.8%, respectively. Of all enrolled patients, 17.4% had an early death. The median survival time was 29.00 months [95% confident interval (CI): 28.22–29.78 months].

**TABLE 1:** Patients’ demographics, clinical characteristics, and therapeutic interventions among bone metastatic breast cancer patients.

Characteristic	Overall	Groups		*p*
		Training cohort	Validation cohort	
n	16,189	14,582	1,607	
Age, mean (SD)	61.67 (14.04)	61.65 (14.04)	61.87 (14.10)	0.547
Sex (%)				0.116
Female	15,998 (98.8)	14,403 (98.8)	1,595 (99.3)	
Male	191 (1.2)	179 (1.2)	12 (0.7)	
Race (%)				0.368
American Indian/Alaska Native	105 (0.6)	92 (0.6)	13 (0.8)	
Asian or Pacific Islander	1,218 (7.5)	1,104 (7.6)	114 (7.1)	
Black	2,356 (14.6)	2,130 (14.6)	226 (14.1)	
White	12453 (76.9)	11201 (76.8)	1,252 (77.9)	
Unknown	57 (0.4)	55 (0.4)	2 (0.1)	
Marital status (%)				0.866
Married (which includes common law)	7,055 (43.6)	6,370 (43.7)	685 (42.6)	
Separated, divorced, or widowed	4,627 (28.6)	4,157 (28.5)	470 (29.2)	
Single (never married)	3,554 (22.0)	3,199 (21.9)	355 (22.1)	
Others	953 (5.9)	856 (5.9)	97 (6.0)	
Geographic areas (%)				0.800
Metropolitan counties	14,345 (88.6)	12,927 (88.7)	1,418 (88.2)	
Nonmetropolitan counties	1,821 (11.2)	1,635 (11.2)	186 (11.6)	
Unknown	23 (0.1)	20 (0.1)	3 (0.2)	
Laterality (%)				0.575
Bilateral, single primary	95 (0.6)	90 (0.6)	5 (0.3)	
Left—origin of primary	7,734 (47.8)	6,970 (47.8)	764 (47.5)	
Only one side—side unspecified	55 (0.3)	48 (0.3)	7 (0.4)	
Paired site, but no information concerning laterality	776 (4.8)	697 (4.8)	79 (4.9)	
Right—origin of primary	7,529 (46.5)	6,777 (46.5)	752 (46.8)	
Brain metastasis (%)				0.179
No	14,443 (89.2)	13,031 (89.4)	1,412 (87.9)	
Unknown	569 (3.5)	504 (3.5)	65 (4.0)	
Yes	1,177 (7.3)	1,047 (7.2)	130 (8.1)	
Liver metastasis (%)				0.609
No	12,043 (74.4)	10,843 (74.4)	1,200 (74.7)	
Unknown	422 (2.6)	375 (2.6)	47 (2.9)	
Yes	3,724 (23.0)	3,364 (23.1)	360 (22.4)	
Lung metastasis (%)				0.823
No	11,422 (70.6)	10,279 (70.5)	1,143 (71.1)	
Unknown	563 (3.5)	506 (3.5)	57 (3.5)	
Yes	4,204 (26.0)	3,797 (26.0)	407 (25.3)	
T stage (%)				0.754
T0	346 (2.1)	313 (2.1)	33 (2.1)	
T1	1,841 (11.4)	1,655 (11.3)	186 (11.6)	
T2	4,219 (26.1)	3,795 (26.0)	424 (26.4)	
T3	2,233 (13.8)	2,004 (13.7)	229 (14.3)	
T4	4,453 (27.5)	4,037 (27.7)	416 (25.9)	
TX	3,097 (19.1)	2,778 (19.1)	319 (19.9)	
N stage (%)				0.132
N0	3,881 (24.0)	3,468 (23.8)	413 (25.7)	
N1	6,894 (42.6)	6,255 (42.9)	639 (39.8)	
N2	1,443 (8.9)	1,297 (8.9)	146 (9.1)	
N3	1,919 (11.9)	1,730 (11.9)	189 (11.8)	
NX	2,052 (12.7)	1,832 (12.6)	220 (13.7)	
Histologic type (%)				0.471
Adenomas and adenocarcinomas	1,080 (6.7)	973 (6.7)	107 (6.7)	
Ductal and lobular neoplasms	13,557 (83.7)	12,205 (83.7)	1,352 (84.1)	
Epithelial neoplasms, NOS	1,075 (6.6)	967 (6.6)	108 (6.7)	
Squamous cell neoplasms	16 (0.1)	14 (0.1)	2 (0.1)	
Unspecified neoplasms	388 (2.4)	352 (2.4)	36 (2.2)	
Others	73 (0.5)	71 (0.5)	2 (0.1)	
Grade (%)				0.624
Grade I	855 (5.3)	767 (5.3)	88 (5.5)	
Grade II	4,170 (25.8)	3,738 (25.6)	432 (26.9)	
Grade III	3,705 (22.9)	3,357 (23.0)	348 (21.7)	
Grade IV	55 (0.3)	51 (0.3)	4 (0.2)	
Unknown	7,404 (45.7)	6,669 (45.7)	735 (45.7)	
ER status (%)				0.193
Borderline/unknown	1,483 (9.2)	1,338 (9.2)	145 (9.0)	
Negative	2,385 (14.7)	2,172 (14.9)	213 (13.3)	
Positive	12,321 (76.1)	11,072 (75.9)	1,249 (77.7)	
PR status (%)				0.867
Borderline/unknown	1,656 (10.2)	1,497 (10.3)	159 (9.9)	
Negative	4,633 (28.6)	4,167 (28.6)	466 (29.0)	
Positive	9,900 (61.2)	8,918 (61.2)	982 (61.1)	
HER_2_ (%)				0.165
Borderline/unknown	2,230 (13.8)	2,001 (13.7)	229 (14.3)	
Negative	10,885 (67.2)	9,784 (67.1)	1,101 (68.5)	
Positive	3,074 (19.0)	2,797 (19.2)	277 (17.2)	
Cancer-directed surgery (%)				0.849
No	12,296 (76.0)	11,077 (76.0)	1,219 (75.9)	
Unknown	274 (1.7)	244 (1.7)	30 (1.9)	
Yes	3,619 (22.4)	3,261 (22.4)	358 (22.3)	
Radiation (%)				0.862
No/unknown	10,725 (66.2)	9,664 (66.3)	1,061 (66.0)	
Yes	5,464 (33.8)	4,918 (33.7)	546 (34.0)	
Chemotherapy (%)				0.104
No/unknown	7,642 (47.2)	6,852 (47.0)	790 (49.2)	
Yes	8,547 (52.8)	7,730 (53.0)	817 (50.8)	
Early death (%)				0.329
No	13,374 (82.6)	12,061 (82.7)	1,313 (81.7)	
Yes	2,815 (17.4)	2,521 (17.3)	294 (18.3)	

SD, standard deviation; T, tumor; N, nodes; NOS, not otherwise specified; ER, estrogen receptor; PR, progesterone receptor; HER_2_, human epidermal growth factor receptor-2.

### Selection of model predictors

Patients from the entire cohort were split into a training group and validation group. Table 1 demonstrates that the two groups were comparable because all variables were similarly distributed between the two groups (All *p*-values were more than 0.10). The selection of the model predictors was performed in the training group.

To begin with, a comparison of the clinical characteristics was performed on the basis of the presence of early death (Table 2). When compared with patients who did not have early death, this study found that patients of early death had older age (*p* < 0.001); a higher proportion of separated, divorced, or widowed marital status (*p* < 0.001); nonmetropolitan counties (*p* = 0.013), paired site laterality (*p* < 0.001), more organ metastasis (*p* < 0.001), a higher rate of T4 stage (*p* < 0.001) and NX stage (*p* < 0.001), a lower rate of ductal and lobular neoplasms (*p* < 0.001), and a higher rate of unknown grade (*p* < 0.001). In addition, early death had a significant lower proportion of ER positive status (*p* < 0.001), PR positive status (*p* < 0.001), and HER2 positive status (*p* < 0.001), and less cancer-directed surgery (*p* < 0.001), radiation (*p* < 0.001), and chemotherapy (*p* < 0.001).

**TABLE 2:** Characteristic comparison of early death among bone metastatic breast cancer patients in the training group.

Characteristic	Overall	Early death		*p*
		No	Yes	
n	14,582	12,061	2,521	
Age [mean (SD)]	61.65 (14.04)	60.42 (13.83)	67.51 (13.53)	<0.001
Sex (%)				0.279
Female	14,403 (98.8)	11,907 (98.7)	2,496 (99.0)	
Male	179 (1.2)	154 (1.3)	25 (1.0)	
Race (%)				0.237
American Indian/Alaska Native	92 (0.6)	79 (0.7)	13 (0.5)	
Asian or Pacific Islander	1,104 (7.6)	925 (7.7)	179 (7.1)	
Black	2,130 (14.6)	1741 (14.4)	389 (15.4)	
White	11,201 (76.8)	9,266 (76.8)	1,935 (76.8)	
Unknown	55 (0.4)	50 (0.4)	5 (0.2)	
Marital status (%)				<0.001
Married (which includes common law)	6,370 (43.7)	5,517 (45.7)	853 (33.8)	
Separated, divorced, or widowed	4,157 (28.5)	3,243 (26.9)	914 (36.3)	
Single (never married)	3,199 (21.9)	2,594 (21.5)	605 (24.0)	
Others	856 (5.9)	707 (5.9)	149 (5.9)	
Geographic areas (%)				0.013
Metropolitan counties	12,927 (88.7)	10,734 (89.0)	2,193 (87.0)	
Nonmetropolitan counties	1,635 (11.2)	1,312 (10.9)	323 (12.8)	
Unknown	20 (0.1)	15 (0.1)	5 (0.2)	
Laterality (%)				<0.001
Bilateral, single primary	90 (0.6)	65 (0.5)	25 (1.0)	
Left—origin of primary	6,970 (47.8)	5,828 (48.3)	1,142 (45.3)	
Only one side—side unspecified	48 (0.3)	39 (0.3)	9 (0.4)	
Paired site, but no information concerning laterality	697 (4.8)	509 (4.2)	188 (7.5)	
Right—origin of primary	6,777 (46.5)	5,620 (46.6)	1,157 (45.9)	
Brain metastasis (%)				<0.001
No	13,031 (89.4)	10,986 (91.1)	2,045 (81.1)	
Unknown	504 (3.5)	367 (3.0)	137 (5.4)	
Yes	1,047 (7.2)	708 (5.9)	339 (13.4)	
Liver metastasis (%)				<0.001
No	10,843 (74.4)	9,421 (78.1)	1,422 (56.4)	
Unknown	375 (2.6)	284 (2.4)	91 (3.6)	
Yes	3,364 (23.1)	2,356 (19.5)	1,008 (40.0)	
Lung metastasis (%)				<0.001
No	10,279 (70.5)	8,872 (73.6)	1,407 (55.8)	
Unknown	506 (3.5)	376 (3.1)	130 (5.2)	
Yes	3,797 (26.0)	2,813 (23.3)	984 (39.0)	
T stage (%)				<0.001
T0	313 (2.1)	257 (2.1)	56 (2.2)	
T1	1,655 (11.3)	1,454 (12.1)	201 (8.0)	
T2	3,795 (26.0)	3,315 (27.5)	480 (19.0)	
T3	2,004 (13.7)	1,739 (14.4)	265 (10.5)	
T4	4,037 (27.7)	3,254 (27.0)	783 (31.1)	
TX	2,778 (19.1)	2,042 (16.9)	736 (29.2)	
N stage (%)				<0.001
N0	3,468 (23.8)	2,824 (23.4)	644 (25.5)	
N1	6,255 (42.9)	5,295 (43.9)	960 (38.1)	
N2	1,297 (8.9)	1,140 (9.5)	157 (6.2)	
N3	1,730 (11.9)	1,528 (12.7)	202 (8.0)	
NX	1,832 (12.6)	1,274 (10.6)	558 (22.1)	
Histologic type (%)				<0.001
Adenomas and adenocarcinomas	973 (6.7)	734 (6.1)	239 (9.5)	
Ductal and lobular neoplasms	12,205 (83.7)	10,489 (87.0)	1,716 (68.1)	
Epithelial neoplasms, NOS	967 (6.6)	629 (5.2)	338 (13.4)	
Squamous cell neoplasms	14 (0.1)	9 (0.1)	5 (0.2)	
Unspecified neoplasms	352 (2.4)	147 (1.2)	205 (8.1)	
Others	71 (0.5)	53 (0.4)	18 (0.7)	
Grade (%)				<0.001
Grade I	767 (5.3)	702 (5.8)	65 (2.6)	
Grade II	3,738 (25.6)	3,265 (27.1)	473 (18.8)	
Grade III	3,357 (23.0)	2,844 (23.6)	513 (20.3)	
Grade IV	51 (0.3)	45 (0.4)	6 (0.2)	
Unknown	6,669 (45.7)	5,205 (43.2)	1,464 (58.1)	
ER status (%)				<0.001
Borderline/unknown	1,338 (9.2)	716 (5.9)	622 (24.7)	
Negative	2,172 (14.9)	1,647 (13.7)	525 (20.8)	
Positive	11,072 (75.9)	9,698 (80.4)	1,374 (54.5)	
PR status (%)				<0.001
Borderline/unknown	1,497 (10.3)	850 (7.0)	647 (25.7)	
Negative	4,167 (28.6)	3,328 (27.6)	839 (33.3)	
Positive	8,918 (61.2)	7,883 (65.4)	1,035 (41.1)	
HER_2_ (%)				<0.001
Borderline/unknown	2001 (13.7)	1,249 (10.4)	752 (29.8)	
Negative	9,784 (67.1)	8,420 (69.8)	1,364 (54.1)	
Positive	2,797 (19.2)	2,392 (19.8)	405 (16.1)	
Cancer-directed surgery (%)				<0.001
No	11,077 (76.0)	8,722 (72.3)	2,355 (93.4)	
Unknown	244 (1.7)	225 (1.9)	19 (0.8)	
Yes	3,261 (22.4)	3,114 (25.8)	147 (5.8)	
Radiation (%)				<0.001
No/unknown	9,664 (66.3)	7,681 (63.7)	1983 (78.7)	
Yes	4,918 (33.7)	4,380 (36.3)	538 (21.3)	
Chemotherapy (%)				<0.001
No/unknown	6,852 (47.0)	4,930 (40.9)	1922 (76.2)	
Yes	7,730 (53.0)	7,131 (59.1)	599 (23.8)	

SD, standard deviation; T, tumor; N, nodes; NOS, not otherwise specified; ER, estrogen receptor; PR, progesterone receptor; HER_2_, human epidermal growth factor receptor-2.

Then, the multivariate analysis demonstrated that older age (*p* < 0.001), single marital status (*p* < 0.001), nonmetropolitan counties (*p* = 0.001), brain metastasis (*p* < 0.001), liver metastasis (*p* < 0.001), lung metastasis (*p* < 0.001), and histologic type of unspecified neoplasms (*p* = 0.006) were significantly associated with more early death (Table 3), while a lower grade (*p* = 0.002), positive ER status (*p* < 0.001), cancer-directed surgery (*p* < 0.001), radiation (*p* < 0.001), and chemotherapy (*p* < 0.001) were significantly protective factors for early death. Significant variables with a *p*-value of less than 0.01 were included to develop models. Finally, 12 variables were selected for modeling.

**TABLE 3:** Multivariate analysis of characteristics for early death among bone metastatic breast cancer patients.

Characteristic	OR	95% CI		*p*
		Lower limit	Upper limit	
(Intercept)	0.071	0.024	0.209	<0.001
Age	1.025	1.021	1.029	<0.001
Sex				
Female	Reference			
Male	0.958	0.594	1.545	0.861
Race				
American Indian/Alaska Native	Reference			
Asian or Pacific Islander	1.391	0.624	3.100	0.419
Black	1.423	0.646	3.134	0.381
White	1.284	0.588	2.803	0.531
Unknown	0.529	0.147	1.904	0.330
Marital status				
Married (which includes common law)	Reference			
Separated, divorced, or widowed	1.145	1.011	1.296	0.033
Single (never married)	1.360	1.188	1.556	<0.001
Others	0.977	0.780	1.223	0.838
Geographic areas				
Metropolitan counties	Reference			
Nonmetropolitan counties	1.312	1.126	1.529	0.001
Unknown	2.914	0.724	11.734	0.132
Laterality				
Bilateral, single primary	Reference			
Left—origin of primary	1.021	0.588	1.774	0.940
Only one side—side unspecified	0.379	0.142	1.009	0.052
Paired site, but no information concerning laterality	0.734	0.408	1.320	0.302
Right—origin of primary	1.037	0.597	1.801	0.897
Brain metastasis				
No	Reference			
Unknown	0.901	0.662	1.226	0.507
Yes	2.326	1.962	2.757	<0.001
Liver metastasis				
No	Reference			
Unknown	1.107	0.773	1.586	0.578
Yes	2.821	2.514	3.165	<0.001
Lung metastasis				
No	Reference			
Unknown	1.057	0.778	1.437	0.723
Yes	1.510	1.352	1.687	<0.001
T stage				
T0	Reference			
T1	0.963	0.654	1.420	0.850
T2	1.082	0.749	1.562	0.675
T3	1.124	0.767	1.646	0.550
T4	1.441	1.001	2.075	0.049
TX	1.272	0.895	1.806	0.179
N stage				
N0	Reference			
N1	0.868	0.762	0.989	0.033
N2	0.891	0.714	1.112	0.308
N3	0.838	0.685	1.025	0.085
NX	1.159	0.983	1.366	0.079
Histologic type				
Adenomas and adenocarcinomas	Reference			
Ductal and lobular neoplasms	0.843	0.697	1.021	0.080
Epithelial neoplasms, NOS	1.249	0.993	1.571	0.058
Squamous cell neoplasms	1.675	0.453	6.202	0.440
Unspecified neoplasms	1.552	1.135	2.122	0.006
Others	0.991	0.531	1.849	0.977
Grade				
Grade I	0.631	0.470	0.846	0.002
Grade II	Reference			
Grade III	1.175	1.004	1.375	0.045
Grade IV	0.754	0.294	1.936	0.557
Unknown	1.158	1.009	1.328	0.037
ER status				
Borderline/unknown	Reference			
Negative	0.795	0.507	1.248	0.318
Positive	0.351	0.229	0.537	<0.001
PR status				
Borderline/unknown	Reference			
Negative	1.679	1.096	2.571	0.017
Positive	1.106	0.726	1.684	0.640
HER_2_				
Borderline/unknown	Reference			
Negative	0.910	0.747	1.108	0.347
Positive	0.861	0.685	1.082	0.199
Cancer-directed surgery				
No	Reference			
Unknown	0.373	0.223	0.623	<0.001
Yes	0.298	0.246	0.361	<0.001
Radiation				
No/unknown	Reference			
Yes	0.623	0.553	0.702	<0.001
Chemotherapy				
No/unknown	Reference			
Yes	0.217	0.192	0.245	<0.001

OR, odds ratio; CI, confident interval; SD, standard deviation; T, tumor; N, nodes, NOS, not otherwise specified; ER, estrogen receptor; PR, progesterone receptor; HER_2_, human epidermal growth factor receptor-2.

### Model development and estimation

**FIGURE 2:**
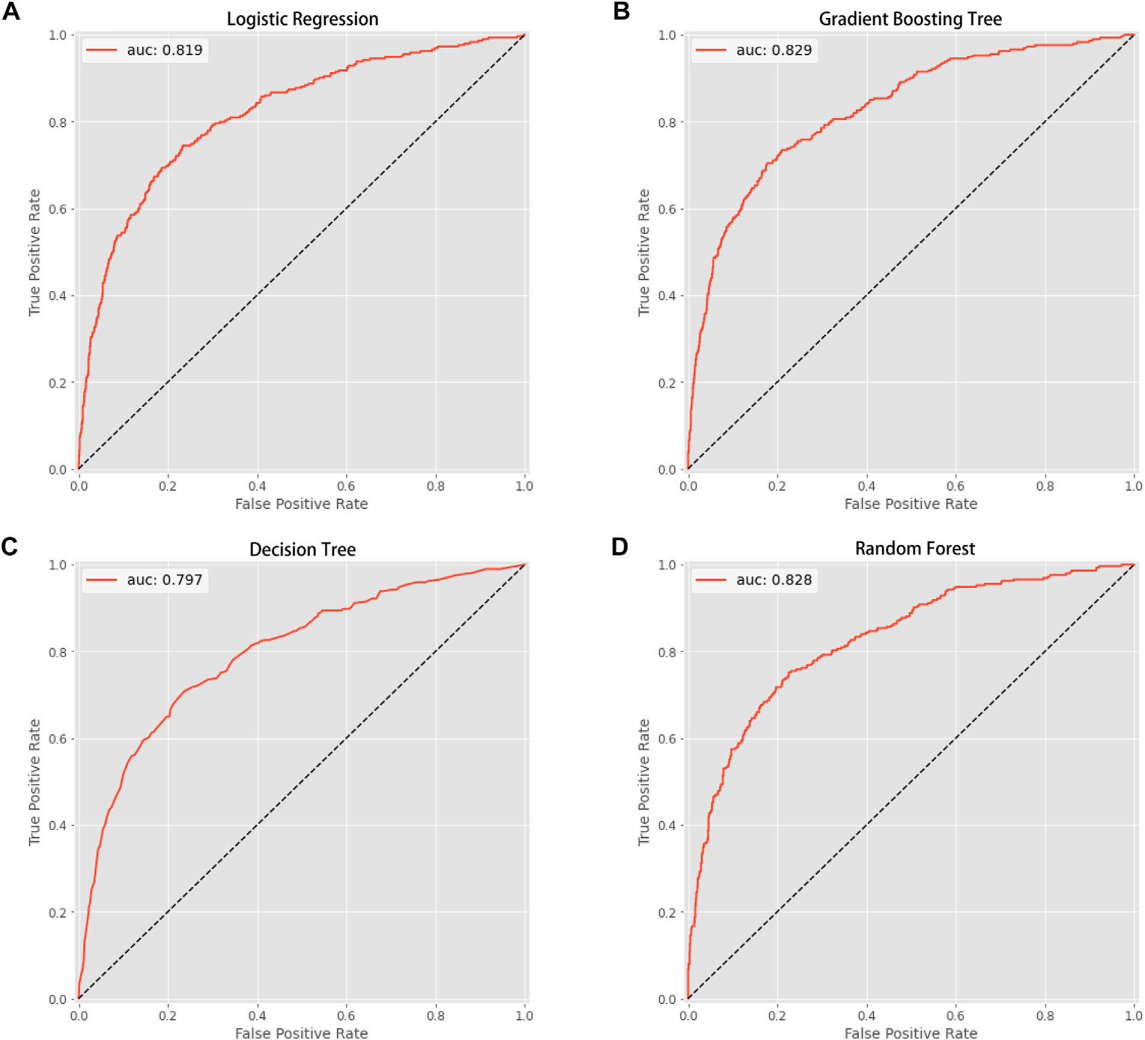
Area under the curve (AUC). **(A)** Logistic regression (AUC value: 0.819); **(B)** gradient boosting tree (AUC value: 0.829); **(C)** decision tree (AUC value: 0.797); **(D)** random forest (AUC value: 0.828).

**FIGURE 3:**
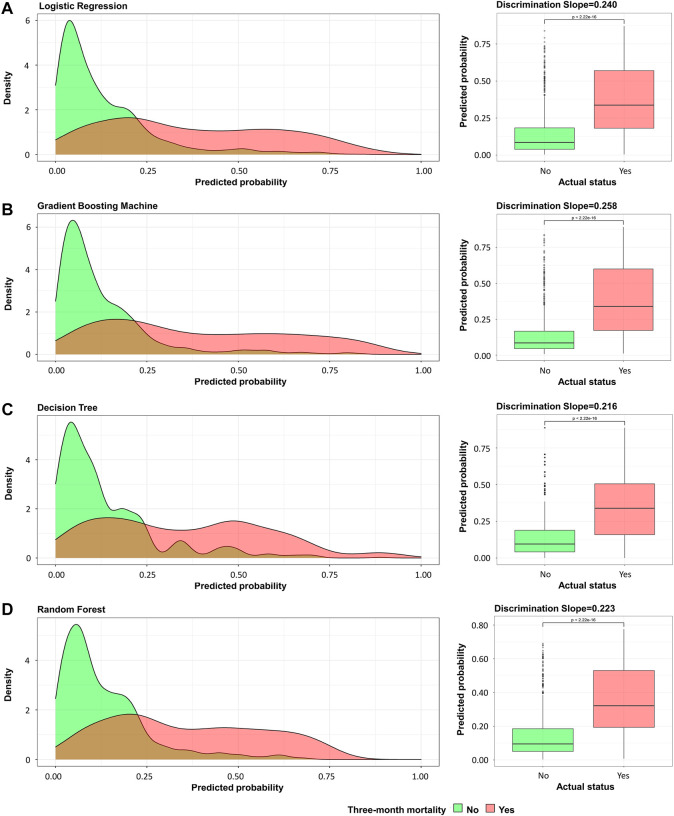
Probability curve and discrimination slope. **(A)** Logistic regression; **(B)** gradient boosting tree; **(C)** decision tree; **(D)** random forest. Green curve indicates patients without early death and red curve indicates patients with early death. Probability curve was plotted with predicted probability of early death against density. On calculating discrimination slope, actual status was plotted against predicted probability.

**FIGURE 4:**
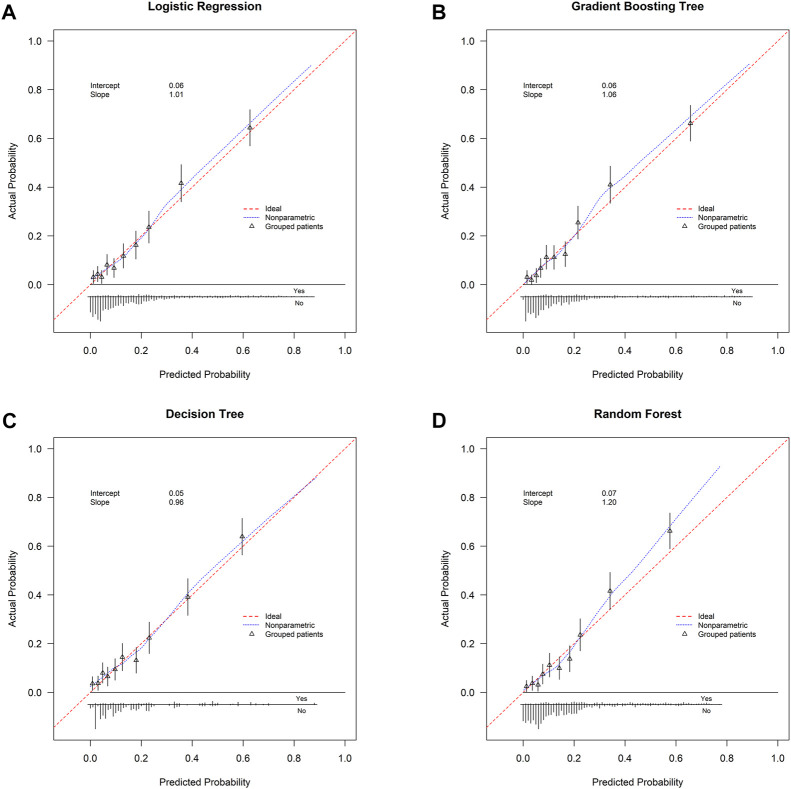
Calibration curve. **(A)** Logistic regression; **(B)** gradient boosting tree; **(C)** decision tree; **(D)** random forest. Calibration curve is plotted with predicted probability against actual probability. Red dotted line indicates ideal consistency between predicted and actual probability of early death. Intercept-in-large value and calibration slope are both shown in the curves.

**FIGURE 5:**
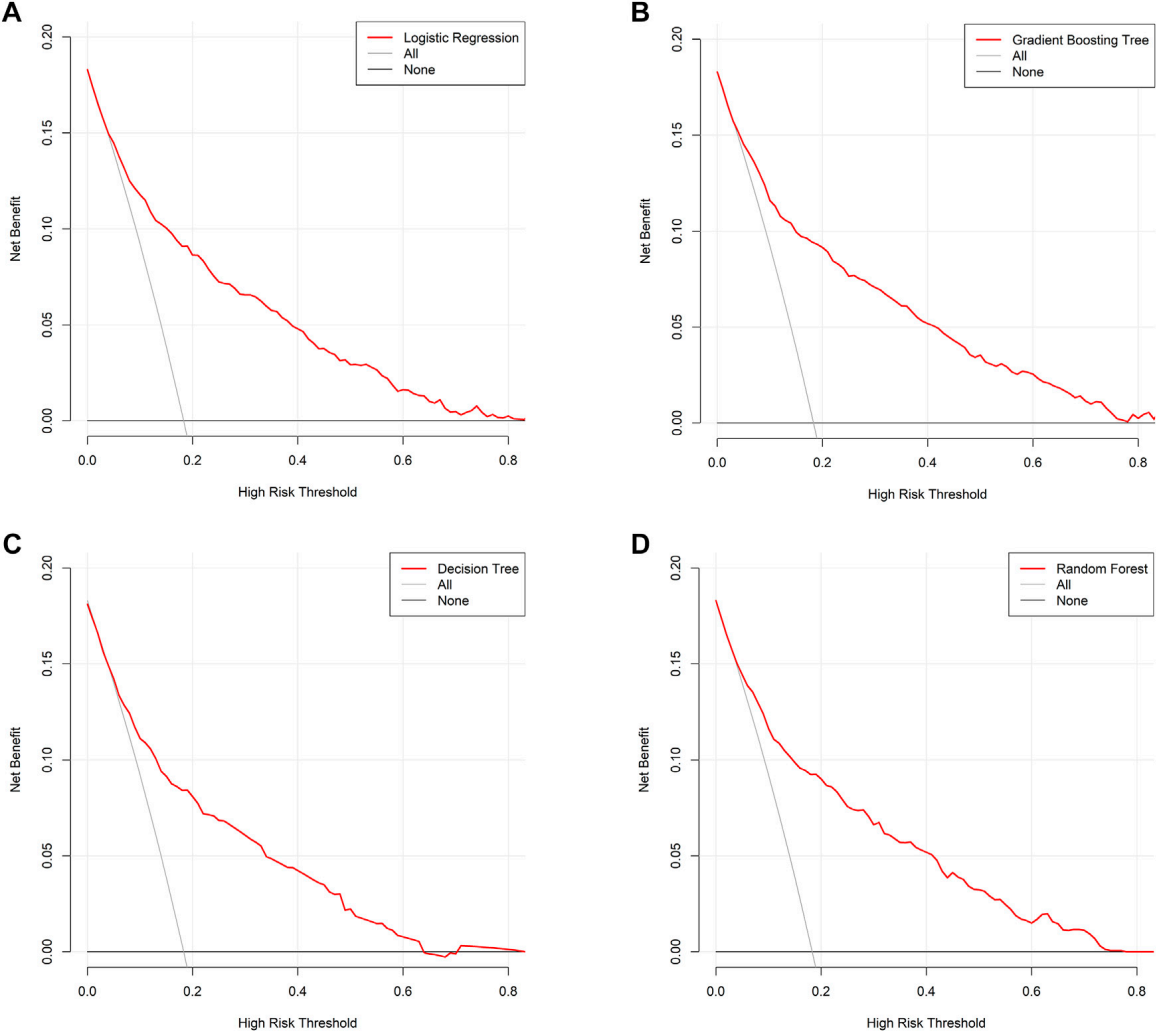
Decision curve analysis. **(A)** Logistic regression; **(B)** gradient boosting tree; **(C)** decision tree; **(D)** random forest. Decision curve is plotted with different risk threshold against net benefit. A larger space between red line and two references indicates more favorable clinical usefulness.

This study used four approaches (the logistic regression, gradient boosting tree, decision tree, and random forest) to develop and optimize models. Supplementary Table S1 summarized the full super-parameter weights of all the four models. Among all the four models, the gradient boosting tree had the highest AUC [0.829, 95% confident interval (CI): 0.802–0.856], and the next highest AUCs were found in the random forest (0.828, 95% CI: 0.801–0.855) and logistic regression (0.819, 95% CI: 0.791–0.847) models (Figure 2). The corresponding accuracy rates of the three models were 0.801, 0.770, and 0.762, respectively (Table 4), and the corresponding discrimination slopes were 0.258, 0.223, and 0.240, respectively (Figure 3). With the lowest overlap and the greatest separation of the two groups in the probability curves, all models, particularly the gradient boosting tree and logistic regression models, had significant separation of patients with and without early death. In addition, the gradient boosting tree model had the lowest Brier score (0.109), followed by the random forest (0.111) and logistic regression (0.112) models. The calibration curves are shown in Figure 4 and decision curves are shown in Figure 5. The above results indicate that the gradient boosting tree model had the optimal predictive performance in comparison to the other models.

**TABLE 4:** Prediction performance of machine learning approaches for predicting early death among bone metastatic breast cancer patients.

Measure	Approach			
	Logistic regression	Gradient boosting tree	Decision tree	Random forest
Mean predicted	0.176	0.176	0.176	0.175
Brier score	0.112	0.109	0.117	0.111
Intercept	0.06	0.06	0.05	0.07
Calibration slope	1.01	1.06	0.96	1.20
AUC (95% CI)	0.819 (0.791–0.847)	0.829 (0.802–0.856)	0.797 (0.767–0.826)	0.828 (0.801–0.855)
Discrimination slope	0.240	0.258	0.216	0.223
Specificity	0.766	0.823	0.764	0.775
Sensitivity (recall)	0.745	0.704	0.707	0.752
NPV	0.931	0.926	0.921	0.933
PPV (precision)	0.416	0.472	0.402	0.427
Youden	1.511	1.527	1.471	1.526
Accuracy	0.762	0.801	0.754	0.770
Threshold	0.191	0.203	0.191	0.193

AUC, area under the curve; CI, confident interval; NPV, negative predictive value; PPV, positive predictive value.

### Model explanation, predictor importance, and risk stratification

**FIGURE 6:**
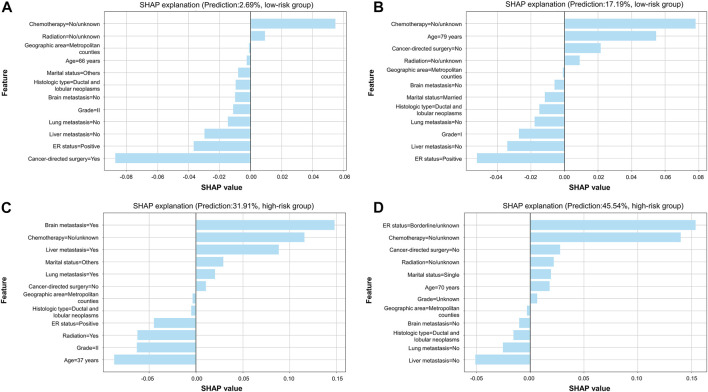
SHAP explanation based on the optimal model. **(A)** Patients with a predicted probability of 2.69% were classified into the low-risk group; **(B)** patients with a predicted probability of 17.19% were classified into the low-risk group; **(C)** patients with a predicted probability of 31.91% were classified into the high-risk group; **(D)** patients with a predicted probability of 45.54% were classified into the high-risk group. In each plot, features are ranked according to importance in individual cases. Every feature can obtain a weight in reference to *x*-axis score. When there is a light-blue bar located at the left of the plot, it denotes that the feature was a protective factor, while that located at the right of the plot represents that the feature was a risk factor. Patients' predicted probability of early death and category of risk groups are shown in the plot.

**FIGURE 7:**
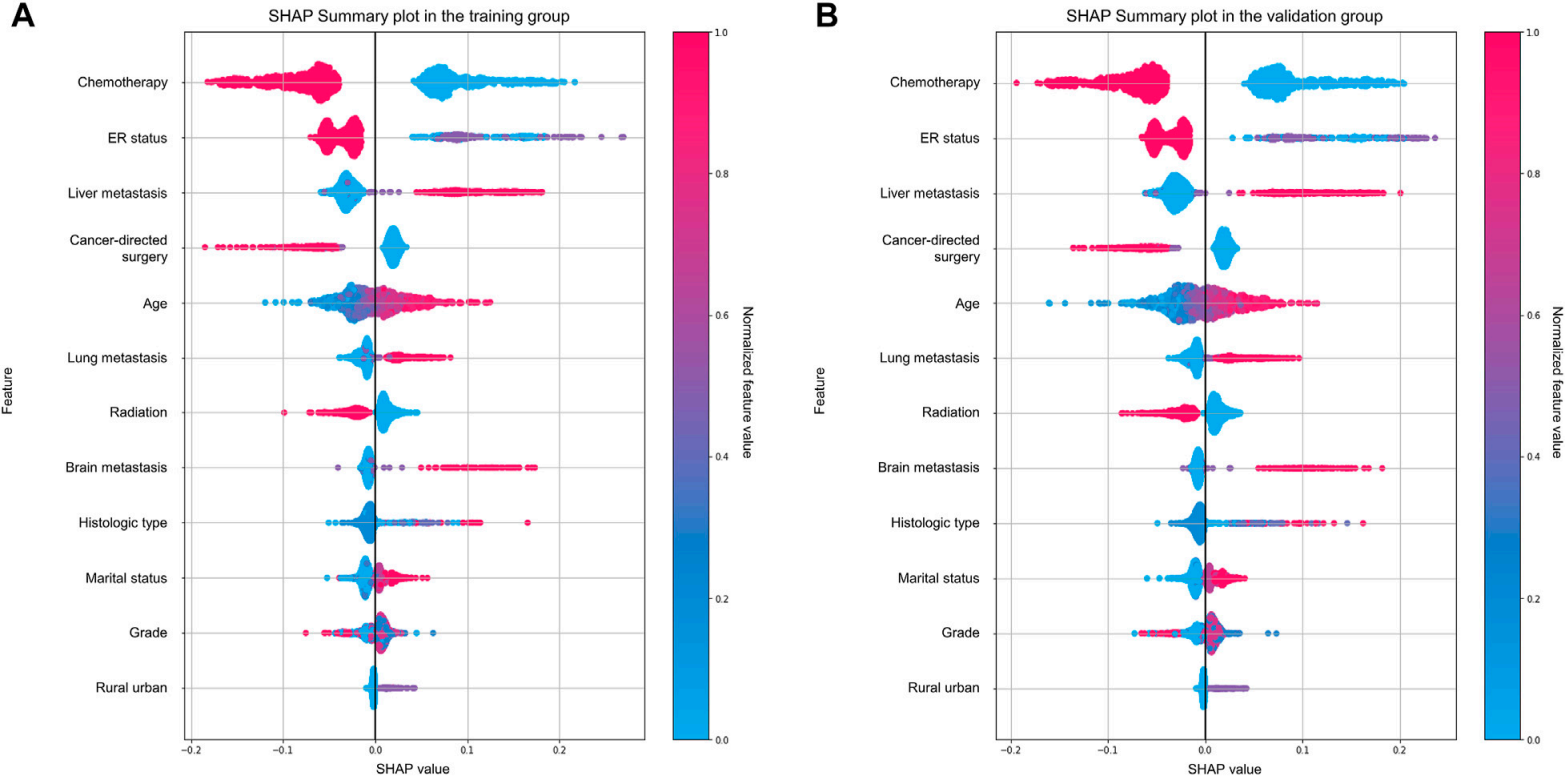
Important analysis of predictors using the SHAP summary plot. **(A)** Training group; **(B)** validation group.

Therefore, model explainability was achieved based on the gradient boosting tree model. Four individual cases were presented in the study to show examples of how to calculate the risk probability of early death and reasons behind it. The first two cases (Figures 6A,B) showed that patients with a low predicted probability of early death survived for more than 3 months (true-negative), while the latter two cases (Figures 6C,D) presented patients with a high predicted probability of early death who died within 3 months (true-positive). The weights of contributing to early death based on the top ten variables in each given case are individually shown in the plots. Figure 7 illustrates the importance of predictors, which demonstrated chemotherapy, ER status, and liver metastasis which were the top three important features in both the training and validation groups.

**FIGURE 8:**
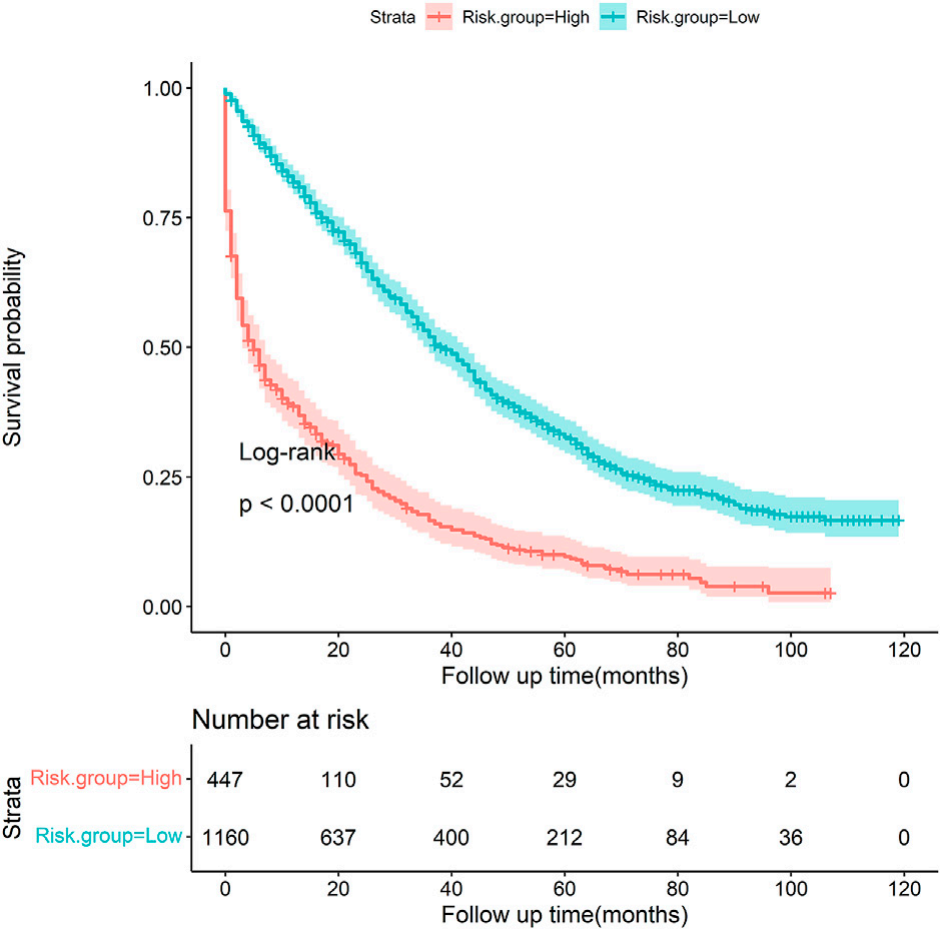
Kaplan–Meier survival curve stratified by risk groups (low *vs*. high, *p* < 0.001, log-rank test).

Based on the optimal threshold (20.00%) from the gradient boosting tree model, patients were categorized into two groups (Table 5). Patients in the high-risk group (46.31%) had a greater six-fold chance of early death than those in the low-risk group (7.50%). Figure 8 shows the Kaplan–Meier survival curve was plotted for patients between the low-risk and high-risk groups, and it demonstrates that patients in the two groups were significantly separated (*p* < 0.001, log-rank test), indicating favorable discrimination.

**TABLE 5:** Risk stratification of patients based on gradient boosting tree.

Risk group	Observed probability (%)	Actual probability (*n* = 1,607)	*p*-value^a^
Low risk (≦20.00%)	8.14	7.50% (87/1,160)	<0.001
High risk (>20.00%)	42.08	46.31% (207/447)	

^a^ indicates a comparison of actual probability between low-risk and high-risk groups using Chi-square test.

## Discussion

This study developed a prediction model to categorize the likelihood of early death specifically among bone metastatic breast cancer patients. In the study, logistic regression and three machine learning models were introduced, and it then examined and compared the four model’s predictive ability to arrive at the best model. The gradient boosting tree model showed the best predictive effectiveness with the lowest Brier score, indicating the best overall predictive performance, and the greatest AUC value and discrimination slope, both of which indicated the best discriminative ability among all models. Along with having the highest specificity, precision, Youden index, and accuracy, the gradient boosting tree model represented the model with the best prediction performance. Therefore, significance of features and risk stratification were both carried out *via* the gradient boosting tree model.

In the present study, the incidence of early death was 17.4% and the median survival time was 29.0 months among all patients. Based on the previous studies, breast cancer patients with bone metastases had a median survival duration of 24.0–30.0 months (Mou et al., 2021; Pan et al., 2021). For these patients, a precise and personalized forecast of survival time is crucial because it can greatly influence the implementation of effective therapy regimens. By contrast, inappropriate estimation of survival could result in over- or under-treating patients.

Currently, certain prediction models have been developed to predict the survival prognosis among bone metastases patients (Forsberg et al., 2011; Ratasvuori et al., 2013; Willeumier et al., 2018; Anderson et al., 2020; Thio et al., 2020; Errani et al., 2021). For example, Thio et al. (2020) used machine learning models to construct and internally test a preoperative survival prediction model of extremity metastatic disease. In the study conducted by Thio et al. (2020), a total of 1,090 patients surgically treated for long bone metastases were included for analysis, and the majority of features that were used to develop the models were laboratory examinations, such as the albumin level, neutrophil-to-lymphocyte ratio, alkaline phosphatase level, hemoglobin level, and calcium level. Although the AUC value for the model was relatively high (0.86) and machine learning models were introduced in this study, the study included many data from laboratory tests and these features might not be easily available to users. Errani et al. (2021) created a prognostic score to choose the best treatment for long bone metastases after analyzing 159 patients with metastatic bone disease who were surgically treated with stable fixation or prosthetic replacement. The prognostic score only included two features: C-reactive protein and tumor diagnosis. Primary tumor was classified into two clinical profiles on the basis of 12-month survival. In the study, after comparing with the other three models, that is, OPTIModel, Scandinavian Sarcoma Group, and PATHFx models (Forsberg et al., 2011; Ratasvuori et al., 2013; Willeumier et al., 2018), the prognostic score had the highest AUC value (0.816). Willeumier et al. (2018) created a prognostic model to predict survival using three independent prognostic features (primary tumor, Karnofsky performance score, and the presence of visceral and/or brain metastases) in 1,520 patients with symptomatic long bone metastases who were treated with orthopedic surgery and/or radiotherapy, and the Harrell C-statistic of this score was only 0.70. In 2011, the PATHFx model was developed by Forsberg et al. (2011) in a cohort of 189 patients who underwent surgery for skeletal metastases. Anderson et al. (2020) updated the PATHFx model in a series of 397 patients in 2020, 189 of whom were originally used to develop the PATHFx model, and the updated model was externally validated in two data sets (*n* = 197 and *n* = 192).

In addition, a number of studies have developed survival prediction models among breast cancer patients. For instance, Han et al. (2021) developed a nomogram to estimate survival outcomes among 17,543 small breast cancer patients using the SEER database, and the nomogram included histologic grade, lymph node stage, estrogen or progesterone receptor status, and molecular subtypes of breast cancer with a C-index of 0.72. Kalafi et al. (2019) used machine learning and deep learning approaches to develop models for predicting survival outcomes among 4,902 breast cancer patients after analyzing 23 clinical variables, and the multilayer perceptron classifier showed the highest accuracy (88.2%).

Of note, the majority of the above-mentioned models were designed inclusively for surgically treated patients with bone metastases after enrolling various primary cancer types or breast cancer patients without bone metastases. Mou et al. (2021) developed a nomogram to predict the overall survival among 145 patients undergoing breast cancer and bone metastasis surgeries after enrolling five clinical characteristics, namely, radiotherapy, pathological type, lymph node metastases, serum alkaline phosphatase, and lactate dehydrogenase. Our study used machine learning to develop models specifically for breast cancer patients with bone metastases, and all the model features were from clinical routine and easily available to orthopedic surgeons and oncologists who could use the model to guide the making of therapeutic strategies for patients with bone metastases. In addition, in the present study, we found that older age, single marital status, nonmetropolitan counties, brain metastasis, liver metastasis, lung metastasis, and histologic type of unspecified neoplasms were risk factors for early death, with a lower grade, positive ER status, cancer-directed surgery, radiation, and chemotherapy being protective factors. The finding suggested that some measures to prevent metastasis in the brain, liver, or lung, clearly determined the histologic type of neoplasms, and treating patients with cancer-directed surgery, radiation, and/or chemotherapy if appropriate would be considerably beneficial for patients' survival prognosis.

Risk classification of patients was accomplished in the study, and patients were split into two risk categories based on the ideal threshold, allowing for the personalized execution of therapeutic strategies. Patients in the high-risk group had above 6.00-time greater odds of early death than those in the low-risk group. Consequently, patients in the high-risk group required more attention. To the author’s knowledge, this study is the first to provide survival prediction models utilizing machine learning techniques exclusively for breast cancer patients with bone metastases. The suggested model raises the performance of nonexpert radiologists and oncologists to that of experts and can be used clinically to predict the survival benefit of breast cancer patients with bone metastases without the need for additional training for staff.

## Limitations

Certain limitations still exist. To begin with, although this study analyzed a variety of potential clinical characteristics, some variables were not incorporated, such as performance status, specific chemotherapy regimens, and laboratory test parameters, due to unavailability in the SEER database. Then, we should be aware that deciding whether to conduct a surgery or not is still a challenging issue and there are other factors to consider when developing treatment plans. Last but not the least, our study offered three machine learning methodologies, and the best model was identified after thoroughly assessing the predicted efficacy of each model. However, the model was not externally tested, necessitating the continued requirement for prospective validation cohorts.

## Conclusion

The gradient boosting tree model demonstrates promising performance with favorable discrimination and calibration in the study, and this model can stratify the risk probability of early death among bone metastatic breast cancer patients. This model may be a pragmatic tool to guide clinical therapeutic strategies and allow information sharing between patients and doctors.

## Data Availability

Publicly available data sets were analyzed in this study. These data can be found at the Surveillance, Epidemiology, and End Results (SEER) database (https://seer.cancer.gov/). This study obtained approval to access the database of the National Cancer Institute in the United States using the reference number 23489-Nov2020.
