# Supplementary material for: A machine learning–Based model to predict early death among bone metastatic breast cancer patients: A large cohort of 16,189 patients
Source: Front Cell Dev Biol. 2022 Dec 7;10:1059597. doi: 10.3389/fcell.2022.1059597 (PMC9768487; doi:10.3389/fcell.2022.1059597)
Supplement: Supplementary file 1 [file Table1.DOCX]

| **Supplementary table 1**. Models and full parameter weights. | |
| --- | --- |
| **Models** | **Full parameter weights** |
| Logistic regression model | LogisticRegression (C=0.1, random_state=42) |
| Gradient boosting tree model | GradientBoostingClassifier (max_depth=2, max_features='auto', min_samples_leaf=57, min_samples_split=82, n_estimators=138, random_state=42) |
| Decision tree model | DecisionTreeClassifier (max_depth=12, max_features='auto', min_samples_leaf=38, min_samples_split=161, random_state=42) |
| Random forest model | RandomForestClassifier (max_depth=42, max_features='log2', min_samples_leaf=25, min_samples_split=80, n_estimators=32, random_state=42) |
