# Supplementary figures and images for: A machine learning–Based model to predict early death among bone metastatic breast cancer patients: A large cohort of 16,189 patients
Source: Front Cell Dev Biol. 2022 Dec 7;10:1059597. doi: 10.3389/fcell.2022.1059597 (PMC9768487; doi:10.3389/fcell.2022.1059597)

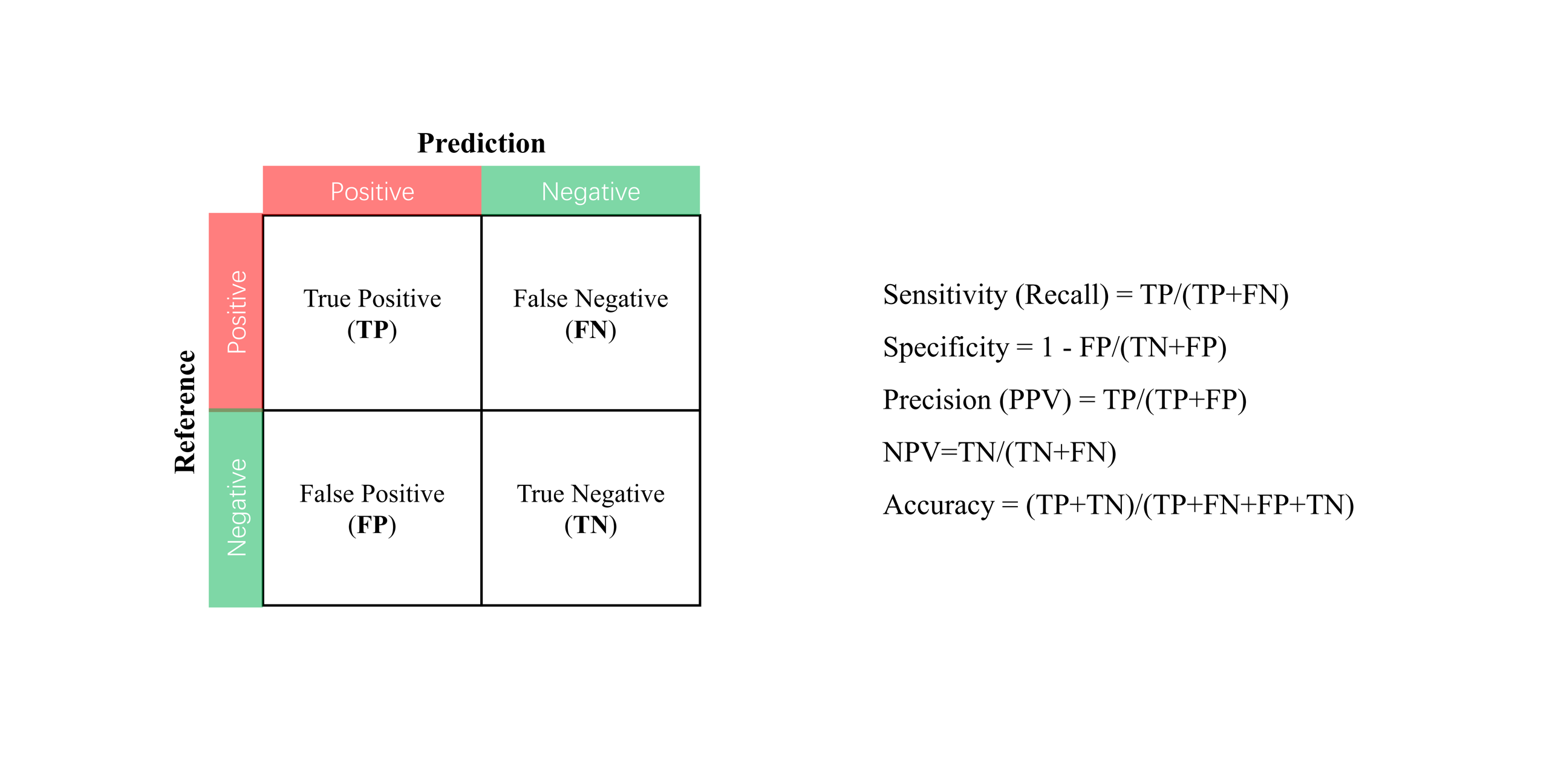

Supplement: Supplementary file 2 [file Image1.TIF]
